# Steven Riley's discussion contribution to papers in Session 3 of the Royal Statistical Society's Special Topic Meeting on COVID‐19 transmission: 11 June 2021

**DOI:** 10.1111/rssa.12981

**Published:** 2022-12-11

**Authors:** Steven Riley

**Affiliations:** ^1^ School of Public Health Imperial College London London UK; ^2^ MRC Centre for Global infectious Disease Analysis Imperial College London London UK

I congratulate Pellis and colleagues, and Dunbar and Held on their excellent papers describing a variety of mechanistic models of SARS‐Cov‐2 transmission, and more generally on their work to support policy formulation during the COVID‐19 pandemic. Both papers address the difficulties of predicting and then evaluating the impact if non‐pharmaceutical interventions (NPIs) against the transmission of severe respiratory pathogens. These are likely to remain key ongoing challenges for the analytical science of pandemic preparedness, with high demand from policy makers for accurate estimates of the epidemiological benefits of NPIs. Here, I would like to make one related methodological point.

There may be benefits in making the null hypotheses in mechanistic modelling studies of NPIs more explicit and more general. For example, models usually contain an underlying basic rate of transmissibility per unit time per infected individual, often denoted β. The parameter β is used to calculate the risk of infection per susceptible and is modified by other parameters to reflect differences in infectiousness, susceptibility and mixing (Keeling & Rohani, 2011). For example, when schools are closed, it may be assumed that mixing patterns for children change on that day and that the efficacy of school closures can be estimated by fitting a version of the model to incidence data which includes a free parameter describing the strength of change in mixing. However, this type of calculation is implicitly making the strong assumption that a step change on the day of the intervention is a good explanation for the overall pattern of changing transmissibility at that time, which may not be the case. It may be useful to explicitly represented β as a smooth function of time in an alternative model, as is common practice for similar parameters in other analytical frameworks (Wood, 2017), so that typical measures of parsimony can be used to assess the information contained in specific model fits when strong assumptions are made about the timing of interventions.
